# Congenital Giant Hydroureteric Cistern in a Duplex System of an Infant

**DOI:** 10.1155/2013/652890

**Published:** 2013-09-19

**Authors:** O. T. Awolaran, L. O. Abdur-Rahman, K. T. Bamigbola, O. M. Adesiyun, A. A. Nasir

**Affiliations:** ^1^Division of Paediatric Surgery, Department of Surgery, University of Ilorin Teaching Hospital, University of Ilorin, P.O. Box 5291, Ilorin 240001, Nigeria; ^2^Department of Radiology, University of Ilorin Teaching Hospital, University of Ilorin, P.O. Box 5291, Ilorin 240001, Nigeria

## Abstract

Duplex collecting system is a congenital genitourinary anomaly commonly found incidentally. Our experience with a duplex system associated with giant hydroureter presenting as mobile abdominal swelling that was noticed from birth, constipation, and failure to thrive is described. Ultrasound and IVU did not assist in making the diagnosis, while a barium enema suggested a colonic duplication. Congenital giant hydroureter should be considered as a differential diagnosis in infants with cystic abdominal swelling. A preserved renal moiety attributed to a dilated ureteric cistern was a unique theory in this case.

## 1. Introduction

A duplex collecting system can be defined as renal units containing two pelvicaliceal systems associated with single (incomplete) or double (complete) ureters. Autopsy studies indicated an incidence of 0.5–1.25% [1, 2]. Over 70% of cases are asymptomatic and are usually detected on imaging procedures for other reasons [3]. Left and right sides are equally affected [4]. Females are affected twice as often as males [3]. In complete duplication, the upper pole ureter usually drains caudal and medial to the lower pole ureter (Weigert Meyer rule) [5, 6].

Common complications of duplex system include vesicoureteric reflux, ureterocele, pelviureteric junction obstruction, and recurrent urinary tract infection. Few cases of hydroureter associated with duplex system have been reported. However, much more uncommon is congenital giant hydroureter with a preserved upper moiety. We report a case of giant hydroureter in a duplex systemwhich was noticed immediately after birth as abdominal swelling. There was associated failure to thrive and constipation.

## 2. Case Report

**Figure 1:**
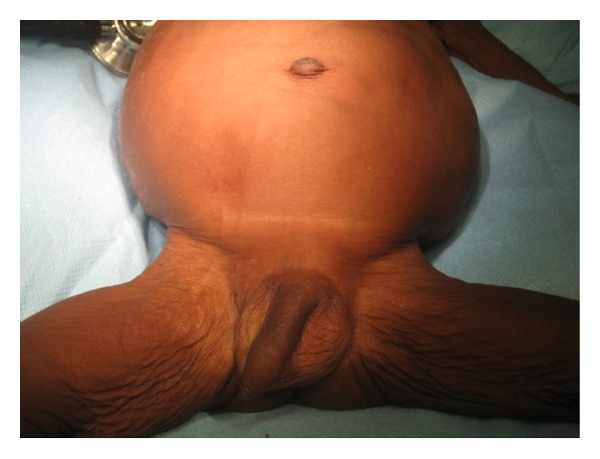
Distended abdomen in a lean baby who weighed 62% of expected weight.

A 4-month-old male presented with abdominal swelling that was noticed at birth. There was failure to gain weight since birth. The swelling involved the whole abdomen but was more on the left and was not progressively increasing in size (Figure 1). There was associated constipation and narrowing of stool calibre. He progressively lost weight since birth despite good appetite. Pregnancy and delivery were not adversely eventful. He passed meconium in the first day of life, and bowel movement has been regular till just around the time of presentation. He was treated repeatedly for urinary tract infection because of recurrent fever. Examination revealed a wasted infant (62% of expected weight) who was febrile and had oral thrush. The abdomen was distended with differential fullness to the left. He had visible bowel markings, and a soft ill-defined mass was palpated on the left flank extending to the left iliac and suprapubic regions. The left testis was nonpalpable in the scrotum or the groin.

**Figure 2:**
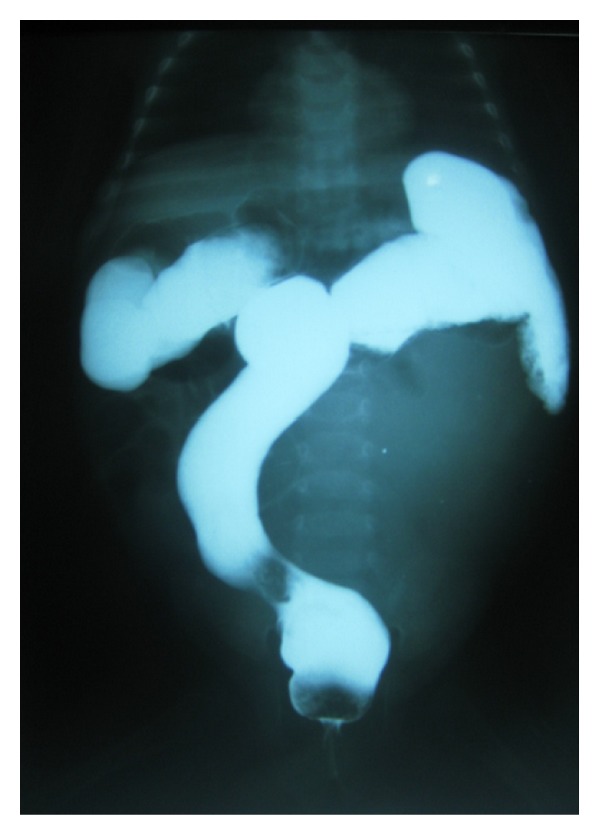
Barium enema showing smooth walled soft tissue shadow that is pushing the sigmoid upwards and to the right.

**Figure 3:**
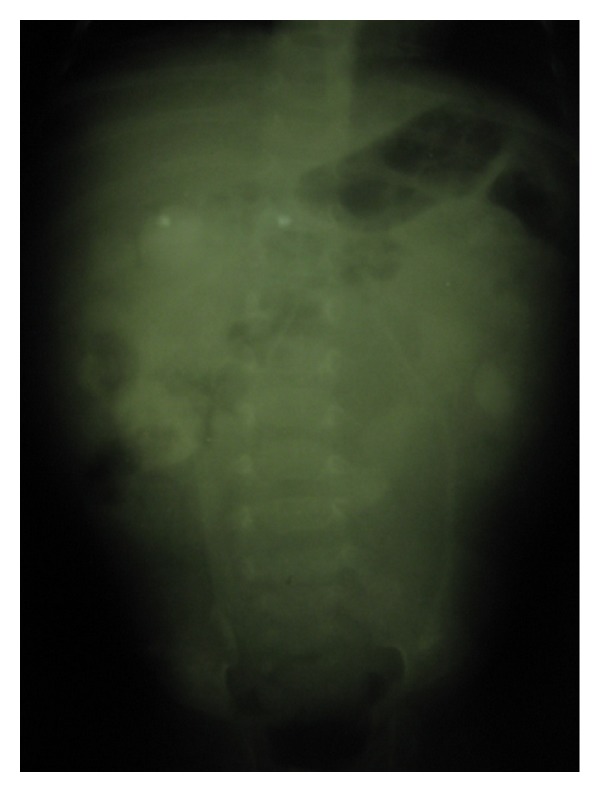
Preoperative intravenous urography showing a central cistern with diluted contrast.

Haemogram, urinalysis, and urine MCS were suggestive of urinary tract infection. Lentiviral screen was negative. Abdominal ultrasound showed huge cystic mass extending from the epigastric region and displacing the bowel loops to the right. An impression of a mesenteric cyst to rule out a gastrointestinal duplication cyst was made. Barium enema showed smooth walled rectum and a soft tissue shadow displacing the sigmoid and descending colon upwards and to the right (Figure 2). Intravenous urography revealed markedly dilated calyces on the left and minimally on the right (Figure 3). Complete outline of the ureters and bladder could not be made out except for a central saccule which is ill defined.

**Figure 4:**
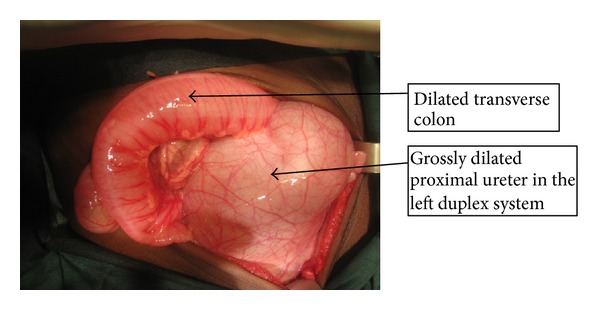
Intraoperative picture showing grossly dilated left proximal ureter in the duplex system (compared with the size of the dilated transverse colon in the picture).

**Figure 5:**
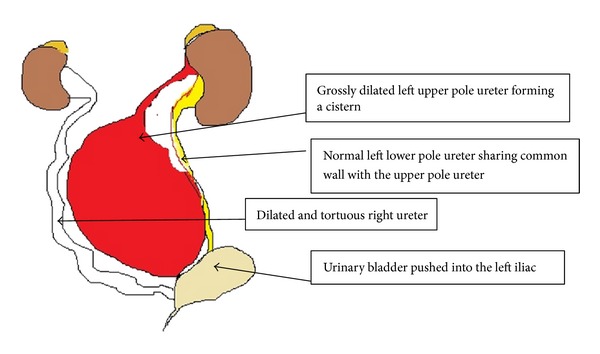
Schematic drawing of the intraoperative findings.

The child was treated with intravenous antibiotics and nutritionally rehabilitated with dextrose and amino acids preparations. At exploratory laparotomy, a complete duplex system was found on the left side. The left kidney was moderately dilated with normal lower pole ureter. There was a huge hydroureter at the mid-third of the upper pole ureter extending from the left lumbar region to the suprapubic and the contralateral right iliac regions displacing the urinary bladder to the ipsilateral iliac fossa (Figure 4). It measured 8 cm × 20 cm and shared a common wall with the lower pole ureter, although there was no communication between the lumina. About 200 mLs of cloudy urine was drained from the dilated segment. It was inserted distally at the bladder neck medial to the normal left ureteric insertion (Figure 5). The left testis was found lying on the wall of the hydroureter. The right ureter was moderately dilated. The proximal segment of the upper pole was functional; hence, excision of the hydroureteric segment with proximal ureteroureterostomy and stent insertion were done. Single staged Fowler-Stephen's left orchidopexy was also done. He had cystoscopy and stent removal 1 week after primary operation, and 2 dilated ureteric orifices and a blind ectopic opening below the right ureteric orifice were seen. Patient has done considerably well by remaining aseptic and progressively gaining weight. Intravenous urography done 4 weeks after operation showed bilateral prompt urine excretion by the kidneys, with minimal residual right ureteric dilatation.

## 3. Discussion

Hydroureter associated with duplex system is not common. Even more uncommon is its occurrence in the neonatal age group causing grossly distended abdomen as seen in this patient. Only very few cases have been reported as in the cases of giant ureter in a duplex system that presented as an abdominal mass in neonate [7, 8]. Our case is a delayed presentation because of ignorance, lack of expertise in the locality of residence of patient, and diagnostic dilemma posed by the pathology to the specialist physicians and surgeons. Similar delay case of a giant hydroureter found incidentally in an adult woman at caesarean section by Mahajan et al. have been reported [9].

Differential diagnoses like mesenteric cyst and enteric duplication cyst were entertained in this infant because of the constipation and radiologic impression. IVU was not helpful because the contrast got diluted in the dilated hydroureteric segment (cistern), hence, the improper outlining of the urinary system. We also think that this cistern also serves as a vent which preserved the upper renal moiety as the majority of the cases of duplex moiety have dysplastic renal poles which necessitates partial or even complete nephrectomy [10, 11]. In this case, CT scan and MRI could have been better diagnostic tools that would define the cystic mass but could not be done due to financial constraints. MAG 3 renal isotopic scan would also assist in confirming the differential function of the renal poles.

Hydroureter associated with duplex system though less common should be entertained as a possible diagnosis in the assessment of infants presenting with recurrent urinary tract infection and soft intra-abdominal mass, especially, where there are limitations to extensive radiological investigations. As seen in this patient, it could be so huge to cause constipation, bladder outlet obstruction, sepsis, and nutritional failure.

Duplex collecting systems are usually asymptomatic and in rare instances may present with hydroureter. This patient is lucky to have a preserved upper pole moiety from the cistern, which we suspect vented the ureter and prevented back pressure effect on the kidney.
